# Loneliness, social isolation and psychiatric disorders: insights from the National Mental Health Survey in Korea

**DOI:** 10.1192/bjo.2025.60

**Published:** 2025-06-19

**Authors:** Eun Jung Kim, Ji Hyun An, Jin Young Jung, Bong Jin Hahm, Jin Pyo Hong

**Affiliations:** Department of Psychiatry, Samsung Medical Center, Sungkyunkwan University School of Medicine, Seoul, South Korea; Department of Psychiatry, Seoul National University College of Medicine, Seoul, South Korea

**Keywords:** Social isolation, loneliness, social support, social network, mental disorders

## Abstract

**Background:**

Loneliness and social isolation pose significant public health concerns globally, with adverse effects on mental health and well-being. Although the terms are often used interchangeably, loneliness refers to the subjective feeling of lacking social connections, whereas social isolation is the objective absence of social support or networks.

**Aims:**

To investigate the prevalence of loneliness and social isolation and their associations with psychiatric disorders.

**Method:**

This study used data from the Republic of Korea National Mental Health Survey 2021, a nationally representative survey. A total of 5511 adults aged 18–79 residing in South Korea participated in the survey. Loneliness and social isolation were assessed using the Loneliness and Social Isolation Scale, whereas psychiatric disorders were evaluated using the Korean version of the Composite International Diagnostic Interview. Multivariate logistic regressions were performed after adjustment for sociodemographic variables.

**Results:**

Among the participants, 11.8% reported experiencing loneliness, 4.3% reported social isolation and 3.4% reported both. Co-occurrence of loneliness and social isolation was significantly associated with psychiatric disorders (adjusted odds ratio (AOR) 7.59, 95% CI: 5.48–10.52). Loneliness alone was associated with greater prevalence and higher probability of psychiatric disorders (AOR 3.12, 95% CI: 2.63–3.71), whereas social isolation did not show any significant association (AOR 0.88, 95% CI: 0.64–1.22).

**Conclusion:**

The co-occurrence of loneliness and social isolation is particularly detrimental to mental health. This finding emphasises the need for targeted interventions to promote social connection and reduce feelings of isolation.

Humans exist within a complex web of relationships, including intimate connections such as family and friends and broader social interactions. These relationships have a profound role in shaping our emotional well-being and, in the long run, have significant implications for our physical health as well.^1^ The landscape of relationship formation has evolved over time. Previously, most relationships were cultivated through face-to-face interactions; however, with the advent of digital media, there has been a notable increase in online platforms facilitating relationship building. The global COVID-19 pandemic has accelerated this shift, prompting individuals to voluntarily or inadvertently distance themselves from traditional social connections and thus exacerbating feelings of loneliness.^2^

Although most previous studies have dealt with loneliness and social isolation as similar concepts, the distinction between loneliness and social isolation is crucial. Loneliness is the subjective experience of distress resulting from perceived inadequacy or dissatisfaction with social connections, whereas social isolation represents an objective state of being disconnected or having limited social networks.^3^ Although these two phenomena may coexist, they can also manifest independently. Individuals experiencing social isolation do not necessarily feel lonely, and, conversely, individuals with extensive social networks may still experience profound loneliness.^4^ Thus, it is imperative to understand the unique characteristics of each of these concepts, particularly when they intersect.

Loneliness is not merely a transient emotion but rather a stable personal trait that can significantly affect long-term psychological well-being.^5,6^ Extensive research, including studies on adolescents, has linked loneliness to future mental health issues, with depression emerging as a consistent concern over time.^7^ Loneliness has also been found to contribute to suicidal behaviour, psychosis and personality disorders.^8–11^ Conversely, individuals with strong social connections demonstrate resilience against psychotic symptoms and have a greater likelihood of recovering from mental health problems.^12^

By contrast, social isolation is associated with immediate negative emotions such as anger and sadness, along with diminished satisfaction with respect to fundamental psychological needs such as self-esteem and cognitive abilities. Affected individuals often adopt self-protective cognitive patterns such as negative interpersonal interactions or heightened negative emotions.^13^ Prolonged social isolation, however, has been linked to heightened risks of depression, cognitive decline and even premature mortality.^14,15^ For individuals with severe mental illness, social isolation negatively affects existing challenges, exacerbating paranoia, impairing insight and decreasing reliance on healthcare services.^16^ Furthermore, individuals resorting to substance use as a negative coping strategy for social isolation tend to exhibit higher levels of isolation.^17^ This intricate relationship is also intertwined with psychological flexibility, with individuals experiencing social isolation having lower levels of this adaptive trait, which further exacerbates their psychological distress.^18^

Despite considerable research on loneliness and social isolation, previous studies have predominantly focused on specific demographic groups, such as youth and the elderly, rather than examining the general population on a nationwide scale. Furthermore, although loneliness and social isolation are known to be linked to psychiatric difficulties including depression, anxiety and suicidal behaviour, these terms are often used interchangeably in the literature. Consequently, there is limited understanding of how the two phenomena affect psychiatric difficulties, both individually and in combination. Given the intertwined yet distinct nature of loneliness and social isolation, it is essential to investigate how they manifest across different age groups and their respective impacts on mental health outcomes. In this study, we aimed to bridge these gaps, thereby offering a more comprehensive understanding of loneliness and social isolation and their implications for mental well-being.

## Method

### Samples

Data were obtained from the Republic of Korea National Mental Health Survey (NMHSK) 2021, which was conducted from June 2021 to August 2021 and was supervised by the National Mental Health Center of the Ministry of Health and Welfare. The NMHSK has been administered every 5 years since 2001 to continuously monitor the current state of mental health in South Korea. The target population is adults between the ages of 18 and 79 years residing in the Republic of Korea and does not include the populations of islands, dormitories, special social facilities or tourist hotels or foreigners residing in the country. The sample was stratified on the basis of the 2019 Population and Housing Census data from Statistics Korea, considering key factors such as city and province, housing type and urban versus rural areas. The validity of these stratification variables was assessed using the prevalence of major depressive disorder (MDD) as a key indicator. Using this approach, the final sample size was determined to be 5500 individuals, ensuring a sampling error of ±1.32 percentage points at a 95% confidence level. The final response rate was 43.8%. In the survey area, one adult was selected from the final selected households using proportional probability sampling and systematic sampling, and a total sample of 5511 people (2757 males and 2754 females) completed the questionnaire. The purpose and method of the survey were explained to all participants, and their written consent was obtained before they participated in the interview. This study was approved by the Samsung Medical Center Institutional Review Board (SMC2022-11-042).

### Assessment of psychiatric disorders

The Korean version of the CIDI (Composite International Diagnostic Interview) 2.1 was used as a diagnostic tool to assess mental health status and sociodemographic variables. The CIDI^19^ is a fully structured tool developed based on the DSM-IV^20^ and ICD-10 criteria.^21^ In many countries, it is widely used as a diagnostic interview to obtain information on the prevalence and correlates of mental disorders in large community samples. The Korean version of the CIDI used in this study was developed and validated by Cho et al^22^ to suit the sociocultural context of South Korea. NMHSK 2021 was conducted using tablet-assisted personal interviewing rather than the previous paper version of K-CIDI to make the interview process more efficient and accurate. For NMHSK 2021, bipolar disorder, schizophrenia spectrum disorder and substance use disorder were excluded owing to their low community prevalence. This study considered lifetime depressive disorders (MDD, dysthymia), anxiety disorders (generalised anxiety disorder (GAD), obsessive–compulsive disorder, social anxiety disorder, agoraphobia and specific phobia), post-traumatic stress disorder (PTSD), alcohol use disorder (AUD), tobacco use disorder (TUD) and any psychiatric disorders. MDD, GAD and specific phobia were separately described owing to their high lifetime prevalence rates.

### Assessment of sociodemographic variables

The demographic characteristics included gender, age, educational level, marital status, occupational status and income as categorical variables. Gender was categorised into male and female. Age was divided into six categories: 18–29 years, 30–39 years, 40–49 years, 50-59 years, 60–69 years and 70–79 years. Educational level was divided into ‘less than 9 years’, ‘10 to 12 years’ and ‘more than 13 years’, according to the participant’s final education level. Marital status was classified into three categories: married; divorced, separated or widowed; and single. Occupational status was categorised as ‘regular worker’, ‘non-regular worker’ and ‘not employed’, and income levels were classified as ‘under $2000’, from $2000 to $4000’, ‘from $4000 to $6000’ and ‘over $6000’. A detailed description of demographic characteristics and other variables collected at NMHSK 2021 can be found elsewhere.^23^

### Assessment of loneliness and social isolation

The Loneliness and Social Isolation Scale was employed in the assessment of participants’ levels of loneliness and social isolation. Originally developed and validated within the Korean population,^24^ this scale was designed to assess both loneliness and social isolation, incorporating elements of social support and social networks. The scale comprises six questions in total, with each component consisting of two items rated on a four-point Likert scale. Participants were assigned to one of four groups: loneliness only group (if their cumulative score for the questions evaluating loneliness was 3 or higher), social isolation only group (if their cumulative score for the questions evaluating social isolation (both social support and social networks) was 4 or higher), loneliness and social isolation group (if they experienced both loneliness and social isolation), or neither lonely nor isolated group (if they did not experience either loneliness or social isolation) (see Supplementary Material 1 available at https://doi.org/10.1192/bjo.2025.60 for details).

### Statistical analysis

Differences in demographic characteristics were analysed using Pearson’s chi-squared tests with the data stratified by participants’ experiences of loneliness and social isolation. Prevalences of psychiatric disorders are presented as percentages based on population and housing census data. A multivariate logistic regression analysis to investigate the associations among loneliness, social isolation and psychiatric disorders was carried out after adjustment for age and gender. Adjusted odds ratios (AOR) are presented with 95% confidence intervals. Standardised weighted values were applied to adjust the skewed distribution, with the final number of participants being 5457. All statistical analyses were conducted using SAS 9.4 for Windows (SAS Institute, Cary, NC, USA) and R package 4.1.3 for Windows (R Foundation for Statistical Computing, Vienna, Austria), with statistical significance set at an alpha level of less than 0.05.

## Results

Table 1 presents the sociodemographic characteristics of participants in the loneliness only, social isolation only, loneliness and social isolation, and neither groups. Among the participants, 11.79% were assigned to the loneliness only group, 4.30% to the social isolation only group, and 3.36% to the loneliness and social isolation group. In the loneliness only group, female individuals (53.7%) were more prevalent than male individuals, whereas males were more prevalent than females in the social isolation only (56.8%) and the loneliness and social isolation (52.6%) groups. Regarding age distribution, the loneliness only, social isolation only, and loneliness and social isolation groups all had higher proportions of middle-aged and elderly participants, whereas the neither lonely nor isolated group displayed a relatively even age distribution. Participants in the loneliness only, social isolation only, and loneliness and social isolation groups were also more likely to have a final education level of middle school diploma or lower, and were more likely to be divorced, separated or widowed compared with those in the neither lonely nor isolated group. Notably, the loneliness and social isolation group had a lower proportion of never-married participants than the other groups. The loneliness and social isolation group also exhibited a notably higher proportion of participants who were unemployed or had a low income (less than $2000) compared with the other groups.

**Table 1:** Sociodemographic characteristics of participants in loneliness and social isolation groups

Sociodemographic characteristics	Loneliness only (*n* = 650)	Social isolation only (*n* = 237)	Loneliness and social isolation (*n* = 185)	Neither lonely nor isolated (*n* = 4440)	*P*-value
Gender, *n* (%)					0.035
Male	301 (46.3)	134 (56.8)	98 (52.6)	2244 (50.5)	
Female	349 (53.7)	102 (43.2)	88 (47.4)	2196 (49.5)	
Age in years, *n* (%)					<0.001
18–29	68 (10.5)	28 (11.7)	9 (5.1)	907 (20.4)	
30–39	86 (13.2)	28 (12.1)	19 (10.1)	752 (16.9)	
40–49	126 (19.3)	36 (15.2)	26 (14.0)	887 (20.0)	
50–59	119 (18.4)	47 (19.8)	48 (25.9)	913 (20.6)	
60–69	159 (24.5)	55 (23.2)	48 (26.0)	665 (15.0)	
70–79	92 (14.1)	43 (18.1)	35 (18.9)	316 (7.1)	
Years of education, *n* (%)					<0.001
≤9	165 (25.4)	71 (30.0)	55 (29.8)	509 (11.5)	
10–12	265 (40.9)	102 (43.2)	87 (46.8)	1810 (40.8)	
≥13	218 (33.7)	63 (26.9)	44 (23.4)	2118 (47.7)	
Marital status, *n* (%)					<0.001
Married	308 (47.4)	145 (61.6)	84 (45.0)	2936 (66.1)	
Divorced, separated or widowed	158 (24.3)	37 (15.7)	66 (35.6)	288 (6.5)	
Single	184 (28.3)	54 (22.7)	36 (19.4)	1216 (27.0)	
Employment status, *n* (%)					<0.001
Employed	291 (44.8)	99 (42.0)	46 (24.6)	2432 (54.8)	
Non-regular worker	135 (20.8)	47 (20.0)	44 (23.9)	798 (18.0)	
Not employed	224 (34.4)	90 (38.1)	96 (51.6)	1210 (27.3)	
Income $, *n* (%)					<0.001
<2000	141 (21.8)	51 (21.6)	65 (35.0)	407 (9.3)	
2000–4000	261 (40.4)	90 (38.1)	58 (31.2)	1353 (30.8)	
4000–6000	177 (27.4)	69 (29.5)	36 (19.3)	1670 (38.0)	
>6000	67 (10.4)	25 (10.8)	27 (14.5)	964 (21.9)	

**Fig. 1:**
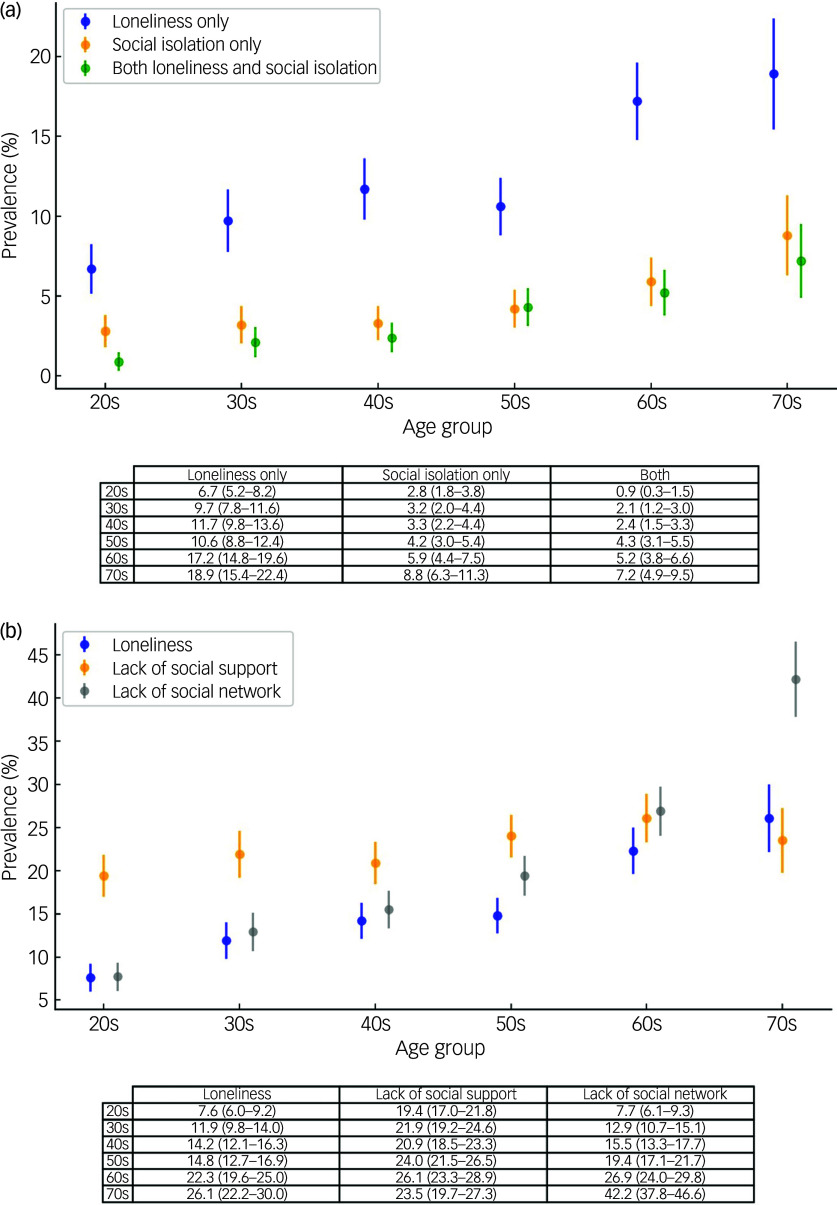
(a) Prevalence of loneliness, social isolation and both by age group (percentages with 95% confidence intervals). Note that the loneliness group excluded participants with social isolation, and the social isolation group excluded those with loneliness. (b) Prevalence of loneliness, lack of social support and lack of social networks by age group (percentages with 95% confidence intervals).

Figure 1(a) presents the prevalence rates of loneliness and social isolation across age groups using a jittered whisker plot. Loneliness and social isolation tended to increase with advancing age. Specifically, loneliness displayed a steady increase, with about 20% of individuals aged 70 years and older reporting loneliness. Social isolation also exhibited a gradual increase with age, albeit with less pronounced effects. The experience of both loneliness and social isolation was most prevalent among individuals in their 70s.

Furthermore, when we analysed social isolation by separately examining social support and social network components (Fig. 1(b)), we found that lower social network scores were more common in older age groups, indicating a trend of increasing social isolation with age. Specifically, 42.2% of individuals aged 70 and older had lower social network scores. By contrast, social support scores remained relatively stable across age groups. This suggests that within the broader construct of social isolation, social networks and loneliness may be more closely associated with age than social support. However, these patterns may also reflect birth cohort differences rather than age-related changes.

Table 2 presents lifetime prevalence rates of psychiatric disorders among the different loneliness and social isolation groups. The prevalence of any psychiatric disorder was highest in the loneliness and social isolation group (69.4%), followed by the loneliness only group (47.7%), with both having significantly higher rates than the social isolation only (21.7%) and the neither lonely nor isolated (23.5%) groups. MDD was the most prevalent psychiatric disorder in both the loneliness and social isolation (40.3%) and the loneliness only (24.9%) groups. Other psychiatric disorders including GAD (9.1%), PTSD (4.3%), AUD (33.5%) and TUD (24.2%) were also most prevalent in the loneliness and social isolation group, followed by the loneliness only group (the exception was specific phobia, for which the prevalence was the same in the loneliness and social isolation group and the loneliness only group). In the case of MDD, GAD and TUD, the prevalence rates in the loneliness and social isolation group were nearly twice as high as those in the loneliness only group. On the other hand, the social isolation only group had even lower prevalence rates of most psychiatric disorders than the neither lonely nor isolated group, although these differences were not statistically significant.

**Table 2:** Lifetime prevalence of common psychiatric disorders in loneliness and social isolation groups^a^

Psychiatric disorder (%)	1. Loneliness only	2. Social isolation only	3. Loneliness and social isolation	4. Neither lonely nor isolated	*P*-value	Group comparison^d^
Major depressive disorder	24.9	3.0	40.3	4.1	<0.001	3 > 1 > 4 > 2
Generalised anxiety disorder	4.2	1.3	9.1	1.0	<0.001	3 > 1 > 2, 4
Specific phobia	9.2	4.3	9.2	5.8	0.001	1 > 4
Post-traumatic stress disorder	4.0	0.8	4.3	1.0	<0.001	1, 3 > 2, 4
Alcohol use disorder	20.0	10.6	33.5	9.5	<0.001	3 > 1 > 2, 4
Tobacco use disorder	12.6	8.1	24.2	8.5	<0.001	3 > 2, 3 > 1 > 4
Anxiety disorder^b^	18.0	6.4	22.6	7.7	<0.001	1, 3 > 2, 4
Any psychiatric disorder^c^	47.7	21.7	69.4	23.5	<0.001	3 > 1 > 2, 4
Any psychiatric disorder						
(excluding alcohol and tobacco use disorder)	33.7	7.7	50.0	10.4	<0.001	3 > 1 > 2, 4

a Weighted for age and gender.

b Anxiety disorder includes obsessive–compulsive disorder, agoraphobia, generalised anxiety disorder, social anxiety disorder and specific phobias.

c Any psychiatric disorder includes major depressive disorder, dysthymia, anxiety disorder, tobacco use disorder and alcohol use disorder.

d Bonferroni correction for multiple comparisons.

Table 3 presents an analysis of odds ratios for lifetime psychiatric disorders in each of the loneliness and social isolation groups. Consistently, the loneliness and social isolation group showed the strongest association with with any lifetime psychiatric disorder (AOR 7.59, 95% CI: 5.48–10.52), followed by the loneliness only group (AOR 3.12, 95% CI: 2.63–3.71). Among lifetime psychiatric disorders, MDD showed the highest associations with the loneliness and social isolation group (AOR 17.77, 95% CI: 12.62–25.03) and the loneliness only group (AOR 8.07, 95% CI: 6.36–10.24). Although the social isolation only group alone did not show any significant association with MDD, there was a significant interaction effect between the loneliness only and the loneliness and social isolation groups (*P* < 0.001), highlighting the importance of considering the joint influence of loneliness and social isolation on depression. Similar indications of a positive interaction between loneliness and social isolation were observed for GAD, PTSD, AUD, TUD and any psychiatric disorder. There was no significant interaction effect between loneliness and social isolation with respect to specific phobia or anxiety disorder. The overall trends remained consistent after additional adjustment for sociodemographic factors including educational level, income and marital status, indicating that adjustments did not alter the findings (as detailed in Supplementary Material 2).

**Table 3:** Associations among loneliness, social isolation and common psychiatric disorders^a^

Psychiatric disorder	Unadjusted odds ratio (95% CI)			Adjusted odds ratio (95% CI)			Interaction effect
	Loneliness only	Social isolation only	Loneliness and social isolation	Loneliness only	Social isolation only	Loneliness and social isolation	*P*-value
Major depressive disorder	7.75 (6.15–9.78)***	0.76 (0.36–1.59)	15.92 (11.46–22.11)***	8.07 (6.36–10.24)***	0.82 (0.39–1.75)	17.77 (12.62–25.03)***	<0.001
Generalised anxiety disorder	4.31 (2.66–7.00)***	1.23 (0.37–4.06)	9.77 (5.46–17.50)***	4.21 (2.57–6.88) ***	1.33 (0.40–4.42)	10.43 (5.72–19.01) ***	0.024
Specific phobia	1.65 (1.23–2.21)**	0.75 (0.40–1.42)	1.67 (1.00–2.78)*	1.61 (1.19–2.18)**	0.82 (0.43–1.56)	1.75 (1.04–2.96)*	0.965
Post-traumatic stress disorder	4.19 (2.57–6.84)***	0.74 (0.16–3.38)	4.44 (2.05–9.58)***	4.28 (2.60–7.03) ***	0.79 (0.17–3.64)	4.81 (2.20–10.54) ***	0.023
Alcohol use disorder	2.38 (1.92–2.96)***	1.14 (0.74–1.74)	4.83 (3.50–6.65)***	2.67 (2.13–3.35)***	1.08 (0.70–1.67)	5.31 (3.77–7.48)***	<0.001
Tobacco use disorder	1.55 (1.21–2.01)**	0.96 (0.60–1.55)	3.47 (2.44–4.93)***	1.75 (1.33–2.30)***	0.83 (0.50–1.35)	3.69 (2.47–5.49)***	0.005
Anxiety disorder	2.65 (2.11–3.33)***	0.79 (0.46–1.36)	3.48 (2.42–4.99)***	2.69 (2.12–3.34)***	0.87 (0.50–1.50)	3.82 (2.62–5.57) ***	0.690
Any psychiatric disorder	2.97 (2.51–3.52)***	0.91 (0.66–1.25)	7.39 (5.37–10.18)***	3.12 (2.63–3.71)***	0.88 (0.64–1.22)	7.59 (5.48–10.52)***	<0.001
Any psychiatric disorder (excluding alcohol and tobacco use disorder)	4.40 (6.64–5.32)***	0.73 (0.45–1.19)	8.62 (6.37–11.68)***	4.62 (3.80–5.63)***	0.81 (0.49–1.32)	10.14 (7.36–13.95)***	0.011

a Compared with the neither lonely nor isolated group, adjusted for age and gender.

*P < 0.05, **P < 0.01, ***P < 0.001.

## Discussion

This study investigated the prevalence and associations of loneliness, social isolation and common psychiatric disorders using nationally representative data in Korea. We found that 11.8% of participants reported loneliness, 4.3% reported social isolation and 3.4% reported both. Our results provide a more comprehensive understanding of the impacts of both subjective loneliness and objective social isolation on mental health compared with previous research in which these were examined separately.

The prevalence rates of loneliness and social isolation vary across countries and depend significantly on the measurement methods and definitions used. A recent comprehensive systematic review reported a prevalence of loneliness ranging from 2.9% to 21.3% across different age groups in Europe.^25^ In Australia, one study found a 17% prevalence of social isolation,^26^ although few studies have specifically addressed social isolation alone in the general population. Consistent with previous findings, the present study also identified notable rates of both loneliness and social isolation, underscoring the global relevance of these issues.^27,28^ Furthermore, despite ongoing debate regarding gender differences in loneliness, our findings corroborate prior evidence indicating that women are more prone to loneliness.^29^ However, when social isolation coincides with loneliness, males tend to exhibit greater susceptibility compared with females.

Subjective feelings of loneliness and lower social network scores were more prevalent in older age groups compared with lower social support scores. Social networks tend to fluctuate across life stages and are influenced by changing roles and responsibilities. Young adults often have larger social networks owing to active engagement in work and social activities, whereas older adults typically experience smaller networks, probably owing to reductions in social interactions following retirement. By contrast, social support scores remained relatively stable across age groups. Social support reflects an individual’s perception of being valued, loved and able to rely on others when needed.^30^ These perceptions are strongly shaped by early life experiences, particularly attachment and belonging in childhood.^31,32^ Prior research suggests that such formative experiences contribute to self-esteem and feelings of being loved,^33^ which tend to remain stable over time. This stability may explain why social support does not exhibit the same age-related variation as social networks and loneliness.

We also found that loneliness and social isolation were closely associated with a range of psychiatric disorders, consistent with previous studies in which these phenomena have been consistently linked to depression, anxiety, suicidal behaviour and heightened levels of stress.^34–36^ Approximately 48% of the loneliness only group had experienced at least one psychiatric disorder in their lifetime, twice as high as the corresponding rate in the neither lonely nor isolated group (24%). In addition, the loneliness only group exhibited significantly increased odds of psychiatric disorders, about 3.1 times that of the neither lonely nor isolated group. However, the social isolation only group did not have significantly elevated prevalence or odds of psychiatric disorders. This finding was in contrast with those of previous research,^37^ which has often treated social isolation as a broader concept encompassing loneliness, without distinguishing between social isolation and loneliness.

Furthermore, the prevalence rates of psychiatric disorders in the social isolation only and neither lonely nor isolated groups appeared to be more influenced by AUD or TUD, decreasing to less than half their previous levels when AUD and TUD were excluded. These groups showed lower associations with affective disorders compared with the loneliness only group or loneliness and social isolation group, yet they remained exposed to the risk of substance use problems. We did not ascertain whether participants in the social isolation only and neither lonely nor isolated groups had undisclosed psychiatric issues; given that individuals often resort to alcohol and nicotine as coping mechanisms for psychological difficulties and maladaptive behaviours,^38^ longitudinal follow-up studies are necessary to monitor these changes.

Our findings suggest that subjective feelings of loneliness are more strongly associated with psychiatric disorders than an objective lack of social relationships. However, given the cross-sectional nature of our study, these associations should not be interpreted as causal. Although loneliness may contribute to psychiatric conditions, psychiatric disorders, particularly chronic ones, may also lead to increased feelings of loneliness and reduced social contacts over time. The mechanisms underlying this relationship remain unclear; however, experiencing social miscognition (feeling lonely despite having sufficient social support and network) may increase vulnerability to psychiatric disorders. This could possibly be elucidated by considering loneliness as a trait of personality, such as neuroticism or borderline personality.^39,40^ In addition, our results indicated that social isolation is not always indicative of maladjustment or maladaptive behaviour leading to psychiatric difficulties; rather, it can be considered to be an active aspect of an individual’s lifestyle. Previous research^41^ has indicated that social isolation does not consistently result in psychological difficulties. However, as we did not measure the spontaneity of isolation, the results of the study must be interpreted with caution.

However, when feelings of loneliness and social isolation co-occur, the impact on mental health may become more profound, as our results suggested significant interaction effects. The loneliness and social isolation group exhibited a prevalence of any psychiatric disorder of approximately 70%, which was 1.5 times higher than that of the loneliness only group and three times higher than that of the neither lonely nor isolated group. In addition, the odds of any psychiatric disorder in the loneliness and social isolation group were up to 7.6 times higher than those of the neither lonely nor isolated group. MDD was the most prevalent psychiatric disorder in the loneliness and social isolation group, with an odds ratio more than double that of the loneliness only group. Similar trends were observed for other psychiatric disorders, including GAD, AUD and TUD. However, anxiety disorders did not exhibit large differences in prevalence and odds between the loneliness only group and the loneliness and social isolation group, suggesting that objective isolation may have a lesser effect on psychological anxiety in these individuals. Participants in the loneliness and social isolation group experienced feelings of loneliness concurrent with a lack of actual social relationships to offset loneliness, and they exhibited greater vulnerability to psychiatric problems than those in the loneliness only group. Although this study did not determine whether social networks mitigate loneliness, the results consistently showed that both the emotional experience of loneliness and an objective lack of social relationships significantly increase the risk of psychiatric disorders beyond expectation. Further research is necessary to investigate the differential impacts of loneliness and social isolation on different types of psychiatric disorder.

To our knowledge, this study represents the first comprehensive and multidimensional assessment of loneliness and social isolation and their associations with psychiatric disorders, providing insight into their individual and combined impacts on a nationwide-level population. Notably, we used an internationally validated diagnostic research tool to assess psychiatric disorders across a broad range of age groups, rather than focusing solely on specific age or gender populations.

Despite its strengths, this study had several limitations. First, its cross-sectional design left open the possibility of reverse causality, wherein loneliness or social isolation could be both a cause and a consequence of psychiatric disorders. Second, owing to their low prevalence, schizophrenia and bipolar spectrum disorders were not included in the NMHSK 2021, necessitating future studies to broaden our understanding to include these disorders. Third, loneliness and social isolation may reflect current circumstances and are subject to change based on an individual’s environment. In particular, this survey was conducted during the COVID-19 pandemic, which may have led to an increase in the prevalence of loneliness and social isolation. Therefore, future research should take into account both trait and state properties of loneliness and social isolation when investigating their associations with psychiatric disorders. Fourth, the NMHSK 2021 employed the previous versions of CIDI and DSM-IV standards, potentially leading to some discrepancies with DSM-5. Fifth, the small sizes of some subgroups, such as those for GAD and PTSD, may have limited statistical power. Last, as this study focused on the Korean population, replication studies are necessary to assess the generalisability of the findings.

Overall, individuals experiencing both loneliness and social isolation demonstrated a heightened association with lifetime psychiatric disorders compared with those experiencing either only loneliness or only social isolation. Whereas loneliness alone was linked to an increased prevalence of psychiatric disorders, social isolation did not exhibit any such association. Furthermore, the prevalence of loneliness increased and the size of social networks decreased with age. Future research should explore various dimensions of loneliness and social isolation to comprehensively understand their causal relationships with psychiatric disorders, giving consideration to methodological variations, age-related factors and cultural contexts.

## Supporting information

Kim et al. supplementary material Kim et al. supplementary material

## Data Availability

This study utilised the Ministry of Health and Welfare’s NMHSK 2021 microdata (NMHSK-15), and the results of the study are not related to the Ministry of Health and Welfare.

## Supplementary material

The supplementary material is available online at https://doi.org/10.1192/bjo.2025.60
