# Supplementary material for: Loneliness, social isolation and psychiatric disorders: insights from the National Mental Health Survey in Korea
Source: BJPsych Open. 2025 Jun 19;11(4):e116. doi: 10.1192/bjo.2025.60 (PMC12188228; doi:10.1192/bjo.2025.60)
Supplement: Kim et al. supplementary material [file S2056472425000602sup001.docx]

**Supplementary material 1.** Loneliness and Social Isolation Scale (LSIS)

| **Loneliness and Social Isolation Scale**  Mark the box which best describes you during the last month. | | | | |
| --- | --- | --- | --- | --- |
| 1. I feel lonely | 0. completely disagree | 1. Somewhat disagree | 2. Somewhat agree | 3. Completely agree |
| 2. I feel isolated | 0. completely disagree | 1. Somewhat disagree | 2. Somewhat agree | 3. Completely agree |
| 3. I can comfortably rely on friends or family. | 3. completely disagree | 2. Somewhat disagree | 1. Somewhat agree | 0. Completely agree |
| 4. There are people who can help me with everyday matters. | 3 completely disagree | 2. Somewhat disagree | 1. Somewhat agree | 0. Completely agree |
| 5. With how many people are you close enough to meet in person at least once a month, or to contact at least once a week? (including family members, relatives, and friends) | 3. 0 | 2. 1~2 | 1. 3~6 | 0. 7 or more |
| 6. On average, how many minutes to you spend speaking with friends or family? (phone, message, or messenger apps) | 3. None | 2. Up to 15 minutes | 1. 15 minutes to 1 hour | 0. More than 1 hour |

*Note*. Loneliness: questions 1 and 2; Social support: questions 3 and 4; Social network: questions 5 and 6.

**Supplementary material 2.** Additional analysis of association between loneliness, social isolation, and common psychiatric disorders

|  | **Model 1 (95% CI)** | | | | **Model 2 (95% CI)** | | | | **Model 3 (95% CI)** | | | **interaction effect** |
| --- | --- | --- | --- | --- | --- | --- | --- | --- | --- | --- | --- | --- |
| **Psychiatric disorder** | **LO** | **SO** | **LS** | **LO** | | **SO** | **LS** | **LO** | | **SO** | **LS** | ***P*-value** |
| Major depressive disorder | 7.75  (6.15-9.78)*** | 0.76  (0.36-1.59) | 15.92  (11.46-22.11)*** | 8.07  (6.36-10.24)*** | | 0.82  (0.39-1.75) | 17.77  (12.62-25.03)*** | 8.71  (6.77-11.21)*** | | 0.88  (0.41-1.86) | 17.47  (12.22-24.98)*** | <0.001 |
| Generalized anxiety disorder | 4.31  (2.66-7.00)*** | 1.23  (0.37-4.06) | 9.77  (5.46-17.50)*** | 4.21  (2.57-6.88) *** | | 1.33  (0.40-4.42) | 10.43  (5.72-19.01) *** | 3.69  (2.20-6.20)*** | | 1.19  (0.36-3.99) | 8.21  (4.33-15.55)*** | 0.024 |
| Specific phobia | 1.65  (1.23-2.21)** | 0.75(0.40-1.42) | 1.67  (1.00-2.78)* | 1.61  (1.19-2.18)** | | 0.82  (0.43-1.56) | 1.75  (1.04-2.96)* | 1.64  (1.20-2.24)** | | 0.81  (0.43-1.54) | 1.75  (1.03-2.97)* | 0.965 |
| Post traumatic stress disorder | 4.19  (2.57-6.84)*** | 0.74(0.16-3.38) | 4.44  (2.05-9.58)*** | 4.28  (2.60-7.03) *** | | 0.79  (0.17-3.64) | 4.81  (2.20-10.54) *** | 3.78  (2.24-6.38)*** | | 0.72  (0.16-3.33) | 3.90  (1.73-8.80)** | 0.023 |
| Alcohol use disorder | 2.38  (1.92-2.96)*** | 1.14  (0.74-1.74) | 4.83  (3.50-6.65)*** | 2.67  (2.13-3.35)*** | | 1.08  (0.70-1.67) | 5.31  (3.77-7.48)*** | 2.94  (2.31-3.74)*** | | 1.13  (0.73-1.75) | 5.71  (3.99-8.17)*** | <0.001 |
| Tobacco use disorder | 1.55  (1.21-2.01)** | 0.96  (0.60-1.55) | 3.47  (2.44-4.93)*** | 1.75  (1.33-2.30)*** | | 0.83  (0.50-1.35) | 3.69  (2.47-5.49)*** | 1.89  (1.42-2.52)*** | | 0.84  (0.51-1.38) | 3.97  (2.61-6.03)*** | 0.005 |
| Anxiety disorder | 2.65  (2.11-3.33)*** | 0.79  (0.46-1.36) | 3.48  (2.42-4.99)*** | 2.69  (2.12-3.34)*** | | 0.87  (0.50-1.50) | 3.82  (2.62-5.57) *** | 2.64  (2.07-3.37)*** | | 0.85  (0.49-1.48) | 3.56  (2.42-5.23)*** | 0.690 |
| Any psychiatric disorder | 2.97  (2.51-3.52)*** | 0.91  (0.66-1.25) | 7.39  (5.37-10.18)*** | 3.12  (2.63-3.71)*** | | 0.88  (0.64-1.22) | 7.59  (5.48-10.52)*** | 3.30  (2.76-3.95)*** | | 0.90  (0.65-1.24) | 7.65  (5.49-10.67)*** | <0.001 |
| Any psychiatric disorder  (excluding alcohol and tobacco use disorder) | 4.40  (6.64-5.32)*** | 0.73  (0.45-1.19) | 8.62  (6.37-11.68)*** | 4.62  (3.80-5.63)*** | | 0.81  (0.49-1.32) | 10.14  (7.36-13.95)*** | 4.77  (3.88-5.86)*** | | 0.83  (0.51-1.35) | 9.76  (7.03-13.55)*** | 0.011 |

compared to the NO group, Model 1 unadjusted, Model 2 adjusted for age and gender, Model 3 adjusted for age, gender, education, marital status and income

**P* < 0.05, ***P* < 0.01, ****P* < 0.001.
